# Psychological Preparedness of Psychologists during the COVID-19 Emergency: Are There Any Individual Differences?

**DOI:** 10.3390/bs14030168

**Published:** 2024-02-23

**Authors:** Sara Veggi, Marialaura Di Tella, Lorys Castelli, Georgia Zara

**Affiliations:** 1 Department of Psychology, University of Turin, 10124 Turin, Italy; sara.veggi@unito.it (S.V.); lorys.castelli@unito.it (L.C.); 2 Department of Law, University of Turin, 10153 Turin, Italy; georgia.zara@unito.it

**Keywords:** psychologists, COVID-19, mental health, psychological preparedness, anxiety, worry

## Abstract

The COVID-19 outbreak has posed an unprecedented global challenge. However, despite the large amount of evidence on the psychological consequences of the pandemic, very few studies have focused on psychologists themselves. (1) Background: The present study aimed to characterise the professional and clinical profile of psychologists facing the COVID-19 outbreak and to investigate the relationship between psychological preparedness and a series of potential predictors (e.g., sociodemographic and professional variables and psychological distress). (2) Methods: A total of 1115 psychologists fully completed an online survey. The data collection period started with the first wave of the pandemic. Participants were asked to provide sociodemographic and professional data and to complete three questionnaires assessing psychological preparedness, worry, and anxiety symptoms. (3) Results: Descriptive results showed that the COVID-19 outbreak did not cause an interruption to the psychological practice of professionals (both in the public and in private sectors) and that psychologists accepted the introduction of technological devices within their regime of work in order to guarantee their service to clients. Only a minority of participants reported clinically significant levels of symptoms of anxiety and worry. Regression analyses revealed that being older, having taken part in training courses on the COVID-19 emergency, and experiencing lower levels of worry and anxiety were all significant predictors of both cognitive and affective psychological preparedness. (4) Conclusions: Taken together, these findings seem to highlight that specific factors can enhance psychological preparedness among psychologists. Therefore, it is vital to inform authorities about the importance of providing emergency programmes to train healthcare workers, especially psychologists, on how to develop psychological preparedness when facing the negative consequences of critical incidents at a universal level, given their crucial role in promoting mental health.

## 1. Introduction

The COVID-19 pandemic has represented an unprecedented global crisis that impacted upon various aspects of individuals’ lives and posed unique challenges to mental health and well-being [1,2]. Extensive research has addressed the psychological vulnerabilities resulting from fear of contagion, grief over loss, social isolation, and economic uncertainties [3,4,5]. In particular, the psychological impact of the pandemic in terms of anxiety, depression, and post-traumatic stress symptoms among healthcare workers has been studied, as they were directly exposed to COVID-19 and strived to understand, mitigate, and cope with its effects [6,7,8,9,10].

While the initial reactive phase of the pandemic focused on the immediate consequences of the contagion, the role of social and psychological sciences became more important in a later, more proactive phase, as they offered a more comprehensive view of how to deal with the long-term effects [11,12].

Although psychological expertise has been integrated into several national policies and action plans [13], very few studies have specifically investigated the effect of the pandemic on psychologists themselves (e.g., [14]). Among these studies, the position of psychologists in supporting the medical system [15] and in responding to the COVID-19 pandemic through research, practice, education, and advocacy [16] has nevertheless been widely recognised.

The pandemic provided psychologists with new challenges and multiple new tasks so they needed to quickly readjust their working patterns in a flexible way. As such, evidence [17] showed that more than one-third of the psychologists reported higher levels of burnout than before the pandemic, especially when they also had young children and worked remotely to guarantee their service. Also, among psychologists who used telepsychology and those who suspended their practice, increased levels of depression were observed when compared to those who continued to work at their workplace; this effect was significantly higher in those psychologists who were single [17]. Similarly, another study [18] has suggested that difficulties in managing both personal and professional life contributed significantly to higher distress for psychologists.

Psychological preparedness is a crucial dimension that affects not only the understanding of dramatic events and disasters but also influences how to respond to them and to the emergency that follows the unexpected. Psychological preparedness can play a key role for psychologists in addressing the many challenges posed by emergency disasters, such as pandemics, and in adapting them to new approaches to mental health care delivery [19].

According to Reser and Morrissey [20], psychological preparedness involves a state of awareness, anticipation, and readiness to respond to an emergency and threatening situation. Research [19,20,21] suggests that the key variables that constitute the foundation of preparedness include both cognitions and emotions. For instance, the degree of knowledge of a determined threatening situation, risk perception, and self-efficacy are based on previous experiences, and gauging the outcome expectancy can help to anticipate some consequences. At the level of the affective domain, self-awareness and emotional regulation involve a sense of perceived responsibility given one’s own professional role and a sense of responsibility for others. It is expected that psychologists have an enhanced sense of psychological preparedness given their training, which develops competence in reading the situations around them and the needs of others. However, it is likely that psychological preparedness is not distributed equally among all psychologists and that some individual differences may affect it.

Starting from these assumptions, the aims of the present study were twofold: first, to characterise the professional and clinical profile of psychologists who were facing the COVID-19 outbreak; second, to investigate the relationship between psychological preparedness and a series of potential predictors (i.e., sociodemographic and professional variables and psychological distress related to worry and anxiety).

## 2. Materials and Methods

### 2.1. Participants and Procedure

The recruitment of potential participants was conducted by an online survey distributed to licensed psychologists through the e-mail addresses officially provided by the Order of Psychologists of Piedmont (Italy). The data collection period coincided with the first wave of the pandemic.

Participation in the study was on a voluntary basis and anonymous, and no compensation was offered. Each participant filled in the survey, which took about 20 min to complete, in one session. The study was approved by the Ethics Committee of the University of Turin (protocol no. 152640). It also follows The Code of Ethics of the World Medical Association (Declaration of Helsinki) for experiments involving humans (2013) and the General Data Protection Regulation (GDPR) (2018).

All participants gave their written informed consent to participate in the study.

A total of 1115 psychologists fully completed the survey, with a response rate of about 14%.

### 2.2. Measures

#### 2.2.1. Sociodemographics and Professional Practice

Participants were asked to indicate their age, gender, presence of children, relationship status, and living conditions. Additionally, they provided information on the length of their professional experience and the degree of job satisfaction. Regarding professional conditions at the time of the COVID-19 pandemic, participants responded to the following items: (1) having continued their professional practice; (2) having been involved in specific initiatives regarding the pandemic; and (3) having used technological devices to provide psychological services and, if so, to what extent technology use improved or worsened their working conditions.

#### 2.2.2. Psychological Measures

- Dunn Worry Questionnaire (DWQ) [22] is a measure of general worry containing 10 items (e.g., “There was little I could do to stop worrying”), with higher scores suggesting a more serious concern. The scale ranged from 0 (“None of the time”) to 4 (“All of the time”), and participants were asked to describe their experience in the previous month. A cut-off score of 21 or above is recommended to identify severe levels of worry. In this sample, Cronbach’s α was 0.83.
- General Anxiety Disorder-7 (GAD-7) [23] is a 7-item anxiety scale (e.g., “Feeling afraid as if something awful might happen”), which detects the frequency and severity of generalised anxiety disorder symptoms. These items have Likert-type responses from 0 (“Not at all”) to 3 (“Nearly every day”). Scores greater than 10 points are indicative of moderate to severe anxiety. In this sample, Cronbach’s α was 0.90.
- Psychological Preparedness for Disaster Threat Scale (PPDTS) [19] is composed of 18 items (e.g., I have a good idea of how I would likely respond in an emergency situation) grouped into two subscales: the Knowledge and Awareness (KA) subscale, referring to cognitive aspects directed at the threat and knowledge of the environment and adaptive responses; and the Anticipation, Awareness, and Management (AAM) subscale, focused on affective aspects involving self-awareness and emotional self-control. Higher scores are suggestive of better psychological preparedness. The scale does not have a cut-off value. The questionnaire has four answer options on a Likert scale ranging from 1 (“Not at all true of me”) to 4 (“Exactly true of me”), with higher scores related to better psychological preparedness. As PPDTS was developed in the context of weather-related and geophysical natural hazard disaster events (e.g., wildfires, floods, and tsunamis), items required adaptation to the COVID-19 emergency. In our sample, Cronbach’s α was 0.84 for the KA subscale and 0.91 for the AAM subscale.

### 2.3. Statistical Analysis

Statistical analyses were performed using the Statistical Package for Social Sciences (SPSS) version 28.0 (IBM SPSS Statistics for Windows, Armonk, NY, USA: IBM Corp.).

Normal distribution was assessed using the indices for asymmetry and kurtosis. All variables were normally distributed.

First, descriptive data were calculated for the entire sample to provide an overview of the participants’ sociodemographic and psychological characteristics. Descriptive data were presented as means with standard deviations for continuous variables or frequencies with percentages for categorical variables.

Second, two hierarchical multiple regression analyses were conducted to assess the relationship between psychological preparedness for the COVID-19 pandemic and a series of potential predictors (sociodemographic variables, professional data, and psychological distress). KA and AAM subscales of the PPDTS were used as dependent variables. Predictor groups were included in the regression model according to the following schema: sociodemographic variables, professional data, and psychological distress (worry and anxiety symptoms).

The enter method was used to include the variables in the predictor groups. Collinearity was assessed using the statistical factor of tolerance and Variance Inflation Factor (VIF).

## 3. Results

### 3.1. Sociodemographics and Professional Practice

Sociodemographic and professional data of the total sample are shown in Table 1.

The sample had a mean age of 43.75 years (SD = 10.73), and most of the participants were women (86.9%; *n* = 969).

In terms of professional practice, the participants had worked as psychologists for an average of 13.36 years (SD = 8.82). Most psychologists continued their professional practice during the pandemic (83.4%; *n* = 930) and relied on technical tools (95.8%; *n* = 1068). These tools included landline or mobile telephony (63.3%; *n* = 706); smartphone video calls (68.3%; *n* = 761); chat messages (e.g., WhatsApp, Telegram) (56.7%; *n* = 632); emails (48.2%; *n* = 537); and conference calls (e.g., Skype, Google Hangouts Meet) (83.5%; *n* = 931). Overall, those for whom the use of technology in their job was a novelty reported a higher workload (42.8%; *n* = 318). Conversely, only 14.8% (*n* = 110) of the sample considered technology to be useful in reducing the workload.

### 3.2. Mental Health during the COVID-19 Emergency

When examining the levels of general problematic worry, a mean score was observed for the DWQ of 12.56 (SD = 6.22), and 11.0% (*n* = 123) of participants reached the cut-off for a severe level of worry [22].

Furthermore, regarding anxiety, participants reported a GAD-7 mean score of 4.53 (SD = 3.03), with 1050 (94.2%) cases of absence or mild symptoms and 65 (5.8%) cases of moderate or severe symptoms.

Focusing on psychological preparedness, participants reported a mean score of 29.00 (SD = 4.12) at the KA subscale and 25.77 (SD = 4.92) at the AAM subscale.

### 3.3. Predictors of Psychological Preparedness: Hierarchical Multiple Regressions

To examine the association between psychological preparedness and a series of potential predictors (demographic variables—age, gender, having children, living conditions; professional data—duration of professional experience, participation in COVID-19 emergency training, continuing to work during the pandemic, technology use; and psychological distress—worry and anxiety symptoms), two hierarchical multiple regression analyses were performed. The KA subscale of the PPDTS was used as a dependent variable in the first regression, while the AAM subscale was used in the second one.

With regard to the KA subscale of the PPDTS, the final model (Model 3) was statistically significant, R^2^ = 0.11, F(10, 1071) = 13.23, *p* < 0.001; adjusted R^2^ = 0.10 (Table 2). In this case, age (*β* = 0.18, *p* < 0.001), having taken part in training courses on the COVID-19 emergency (*β* = 0.09, *p* = 0.002), worry (*β* = −0.13, *p* = 0.004), and anxiety symptoms (*β* = −0.13, *p* = 0.004) were all significant contributors to the final model.

As far as the AAM subscale was concerned, the final model (Model 3) was statistically significant, R^2^ = 0.28, F(10, 1071) = 42.22, *p* < 0.001; adjusted R^2^ = 0.28 (Table 3). Significant predictors of psychological preparedness (AAM subscale) were age (*β* = 0.09, *p* = 0.040), having taken part in training courses on the COVID-19 emergency (*β* = 0.07, *p* = 0.008), worry (*β* = −0.30, *p* < 0.001), and anxiety symptoms (*β* = −0.23, *p* < 0.001).

In both regression analyses, the statistical factor of tolerance and VIF showed that there were no interfering interactions between the variables.

## 4. Discussion

The current study aimed to pursue two main objectives. First, to characterise the clinical and professional profile of psychologists who were facing the COVID-19 outbreak. Second, to examine possible predictors of psychological preparedness (both in its affective and cognitive dimensions).

Regarding the first goal, results highlight that only a minority of psychologists reported clinically significant symptoms of anxiety and worry. These results are in line with previous evidence (e.g., [24]) and can be explained by considering several factors. First, healthcare professionals often operate in diverse and demanding settings, with frontline workers, including those dealing directly with COVID-19 patients in specialised wards, intensive care units, and subintensive wards, reporting higher levels of mental health symptoms. Previous findings have emphasised elevated risks of burnout and vicarious traumatisation among frontline workers compared to their second-line counterparts [6,8,25]. Moreover, among those healthcare professionals who manifested clinically relevant symptoms, the need for psychological support was more frequently expressed [10]. Second, as Humer and colleagues [24] have suggested, psychologists may be more accustomed to handling stressful situations due to their expertise with mentally distressed individuals and can therefore rely on more resilience mechanisms and adaptive coping strategies.

Furthermore, the present study showed that most psychologists continued their professional practice (e.g., psychological support of patients, psychotherapy, writing expert witness reports) even during the most difficult periods of the COVID-19 pandemic, which required rearranging the methodology in adherence to the security procedure required and resorting to technology. This is in line with the findings of Cerasa and colleagues [26] that have shown a massive conversion from in-office to online practice to ensure the continuation of healthcare and psychological services. However, as previously observed by other studies [17,18], technology did not seem to be an asset for professional practice during the emergency, as it was perceived as burdensome on the workload by several psychologists in this sample. It seems relevant to consider that the use of technology devices in psychological practice (e.g., telepsychology) is, in fact, still under critical scrutiny [27] not only because of issues related to privacy and data security but because some still argue that technology may constitute an interference with the purity of the therapeutic setting and professional alliance. However, COVID-19 has obliged humanity to reorganise their lives and forced the readiness of professionals to rearrange their methods to carry on with their duties to protect, support, and inform, which are essential parts of their professional responsibilities.

As far as the second aim of this study was concerned, we found that those who were older and took part in training courses on the COVID-19 emergency were more prepared to face the COVID-19 pandemic than those who were younger and did not participate in training courses. Also, higher levels of psychological distress (i.e., worry and anxiety symptoms) were associated with lower cognitive and affective psychological preparedness. These findings extend those of previous studies [28,29,30], which have investigated potential predictors of psychological preparedness for different disaster experiences among healthcare professionals. For instance, Gandhi and colleagues [29] found that self-efficacy, optimism, and resilience significantly predicted psychological preparedness for COVID-19 in a sample of nursing students. Similarly, the study of Said and colleagues [31] revealed the presence of significant associations between psychological preparedness for disasters and a series of professional (e.g., past training related to disasters, years of experience, and multiple disasters already faced) and psychological variables (e.g., self-efficacy, self-esteem, dispositional optimism, trait anxiety, and post-traumatic stress symptoms) in nurses. Taken together, those findings seem to highlight that enhanced professional competencies and mental health conditions are important factors that can help healthcare workers, including psychologists, to manage emergency crises from both a cognitive and affective perspective.

This study also has some limitations that should be acknowledged. First, while consistent with the gender distribution of psychologists in Italy (who are mostly females, approximately 84%), our sample included a limited number of male participants. Second, we used only self-report measures for the assessment of psychological characteristics. Finally, the present study has adopted a cross-sectional design, which does not allow for causal directions to be drawn. Longitudinal studies with more heterogeneous samples are needed to better clarify the psychological and professional consequences of the COVID-19 pandemic on psychologists and also to distinguish them by their different levels of exposure to COVID-19.

Despite these limitations, the current study represents one of the few attempts to investigate the preparedness of psychologists in light of the COVID-19 outbreak. It is in the nature of any emergency that it occurs without warning, and the health emergency triggered by COVID-19 has deeply shaken the sense of responsibility of professionals who play a key role in promoting and protecting the mental and physical health of individuals, groups, and the social community.

This is why the focus of this study was to understand whether, and to what extent, psychologists were prepared to face COVID-19 in order to continue their supportive and promotive work with their clients and patients, despite the disruption and the sense of social insecurity caused by the emergency. While a great deal of attention has been paid to healthcare workers directly involved in containing and addressing the consequences of the pandemic, less consideration has been given to the psychological snowball effect of COVID-19 on other categories such as psychologists and the extent to which they were prepared. Our results suggest that specific factors were more likely to be associated with psychological preparedness (both cognitive and affective dimensions) for this category of health professionals (i.e., psychologists). In other words, those psychologists who manifested good mental health conditions and were more and better informed about the COVID-19 emergency were found to be more psychologically prepared to face the pandemic, cognitively and emotionally.

## 5. Conclusions

Taking these results together, two points seem to be important as a message to take home. First, psychologists proved to be a professional group that was able to respond to the risk of the COVID-19 emergency with an increased work ethic. In particular, the older and probably more experienced psychologists relied on their psychological preparedness to ensure their service despite the unprecedented health emergency. This was reflected in their willingness to revise their methods and settings (e.g., use of new technologies, digital platforms, telephone methods) and to be there for their patients. Secondly, authorities, professional associations, and societies should be informed about the importance of including specific and mandatory courses on emergency psychology within the training programmes for psychologists. In this way, psychologists can be prepared for the negative consequences of critical incidents at a universal level.

## Figures and Tables

**Table 1:** Sociodemographic characteristics, professional data, and psychological assessment of the total sample (*N* = 1115).

	Mean (SD)	*n* (%)	Range
***Sociodemographic information***			
Age (years)	43.75 (10.73)		22–80
Gender			
*Female*		969 (86.9)	
*Male*		146 (13.1)	
Children			
*Yes*		558 (50.0)	
*No*		557 (50.0)	
Relationship status			
*Single*		154 (13.8)	
*In a relationship*		113 (10.1)	
*Cohabitant*		288 (25.8)	
*Married*		481 (43.1)	
*Separated/Divorced*		71 (6.4)	
*Widower*		8 (0.7)	
Living arrangement			
*Alone*		173 (15.5)	
*With someone (family, partner, friends)*		942 (84.5)	
***Professional data***			
Duration of professional experience (years)	13.36 (8.82)		0–36
Job satisfaction			
*Low*		206 (18.5)	
*Medium*		572 (51.3)	
*High*		337 (30.2)	
Continuation of professional practice during COVID-19			
*Yes*		930 (83.4)	
*No*		185 (16.6)	
Training courses on COVID-19			
*Yes*		627 (56.2)	
*No*		488 (43.8)	
Use of technology in professional practice			
*Yes*		1068 (95.8)	
*No*		47 (4.2)	

**Table 2:** Hierarchical multiple regressions predicting PPDTS KA subscale scores from sociodemographic, professional, and psychological variables (N = 1082).

Predictor Variables	B	*β*	t	95% CI	Adj R^2^	F	ΔR^2^	ΔF
*PPDTS KA*								
***Model 1***					0.05	14.00 ***	0.05	14.00 ***
Age	0.09	0.23	7.07 ***	0.06; 0.11				
Gender	0.23	0.02	0.62	−0.49; 0.95				
Children	−0.36	−0.04	−1.29	−0.91; 0.19				
Living arrangement	0.16	0.01	0.45	−0.55; 0.87				
***Model 2***					0.05	8.30 ***	0.01	2.52 *
Age	0.08	0.21	4.23 ***	0.04; 0.11				
Gender	0.28	0.02	0.76	−0.44; 1.00				
Children	−0.38	−0.05	−1.32	−0.94; 0.19				
Living arrangement	0.17	0.02	0.48	−0.54; 0.88				
Duration of professional experience	0.01	0.02	0.37	−0.04; 0.05				
Training courses on COVID-19	0.67	0.08	2.67 **	0.18; 1.16				
Use of technology in professional practice	0.73	0.04	1.15	−0.52; 1.98				
Continuation of professional practice during COVID-19	0.04	0.00	0.10	−0.65; 0.73				
***Model 3***					0.10	13.23 ***	0.05	31.06 ***
Age	0.07	0.16	3.66 ***	0.03; 0.10				
Gender	0.012	0.00	0.04	−0.69; 0.72				
Living arrangement	0.07	0.01	0.19	−0.63; 0.76				
Duration of professional experience	−0.01	−0.01	−0.23	0.05; 0.04				
Training courses on COVID-19	0.74	0.09	3.03 **	0.26; 1.22				
Use of technology in professional practice	0.63	0.03	1.01	−0.59; 1.84				
Continuation of professional practice during COVID-19	−0.04	−0.00	−0.11	−0.71; 0.64				
DWQ Total	−0.08	−0.13	−2.91 **	−0.14; −0.03				
GAD-7 Total	−0.17	−0.13	−2.92 **	−0.29; −0.06				

PPDTS = Psychological Preparedness for Disaster Threat Scale; KA = Knowledge and Awareness; DWQ = Dunn Worry Questionnaire; GAD-7 = General Anxiety Disorder. * *p* < 0.05; ** *p* < 0.01; *** *p* < 0.001. Note: Data regarding some variables were missing, and this explains why the sample included in the regression models is smaller. As far as the AAM subscale was concerned, the final model (Model 3) was statistically significant, R^2^ = 0.28, F(10, 1071) = 42.22, *p* < 0.001; adjusted R^2^ = 0.28 (Table 3). Significant predictors of psychological preparedness (AAM subscale) were found to be age (*β* = 0.09, *p* = 0.040), having taken part in training courses on the COVID-19 emergency (*β* = 0.07, *p* = 0.008), worry (*β* = −0.30, *p* < 0.001), and anxiety symptoms (*β* = −0.23, *p* < 0.001). In both regression analyses, the statistical factor of tolerance and VIF showed that there were no interfering interactions between the variables.

**Table 3:** Hierarchical multiple regressions predicting PPDTS AAM subscale scores from sociodemographic, professional, and psychological variables (N = 1082).

Predictor Variables	B	*β*	t	95% CI	Adj R^2^	F	ΔR^2^	ΔF
*PPDTS AAM*								
***Model 1***					0.04	13.05 ***	0.05	13.05 ***
Age	0.09	0.21	6.21 ***	0.06; 0.12				
Gender	0.91	0.06	2.08 *	0.05; 1.77				
Children	−0.20	−0.02	−0.58	−0.85; 0.46				
Living arrangement	0.38	0.03	0.88	−0.47; 1.22				
***Model 2***					0.05	7.36 ***	0.01	1.65
Age	0.07	0.15	3.10 **	0.03; 0.11				
Gender	1.01	0.07	2.29 *	0.14; 1.87				
Children	−0.25	−0.03	−0.73	−0.93; 0.42				
Living arrangement	0.38	0.03	0.88	−0.47; 1.23				
Duration of professional experience	0.04	0.06	1.25	−0.02; 0.09				
Training courses on COVID-19	0.51	0.05	1.71	−0.08; 1.10				
Use of technology in professional practice	0.95	0.04	1.25	−0.54; 2.44				
Continuation of professional practice during COVID-19	−0.44	−0.03	−1.05	−1.26; 0.38				
***Model 3***					0.28	42.22 ***	0.23	172.26 ***
Age	0.04	0.09	2.06 *	0.00; 0.08				
Gender	0.34	0.02	0.89	−0.41; 1.10				
Children	−0.25	−0.03	−0.83	−0.84; 0.34				
Living arrangement	0.12	0.01	0.31	−0.62; 0.85				
Duration of professional experience	0.00	0.00	0.03	−0.05; 0.05				
Training courses on COVID-19	0.69	0.07	2.67 **	0.18; 1.21				
Use of technology in professional practice	0.73	0.03	1.10	−0.57; 2.03				
Continuation of professional practice during COVID-19	−0.63	−0.05	−1.73	−1.35; 0.09				
DWQ Total	−0.24	−0.30	−7.79 ***	−0.30; −0.18				
GAD-7 Total	−0.38	−0.23	−5.90 ***	−0.50; −0.25				

PPDTS = Psychological Preparedness for Disaster Threat Scale; AAM = Anticipation, Awareness and Management; DWQ = Dunn Worry Questionnaire; GAD-7 = General Anxiety Disorder. * *p* < 0.05; ** *p* < 0.01; *** *p* < 0.001. Note: data regarding some variables were missing, and this explains why the sample included in the regression models is smaller.

## Data Availability

The data that support the findings of this study are available on reasonable request from the corresponding author.

## References

[1] Esterwood E., Saeed S.A. (2020). Past Epidemics, Natural Disasters, COVID19, and Mental Health: Learning from History as we Deal with the Present and Prepare for the Future. Psychiatr. Q..

[2] Jin Y., Sun T., Zheng P., An J. (2021). Mass quarantine and mental health during COVID-19: A meta-analysis. J. Affect. Disord..

[3] Levine R.L. (2022). Addressing the Long-term Effects of COVID-19. JAMA.

[4] Negri A., Conte F., Caldiroli C.L., Neimeyer R.A., Castiglioni M. (2023). Psychological Factors Explaining the COVID-19 Pandemic Impact on Mental Health: The Role of Meaning, Beliefs, and Perceptions of Vulnerability and Mortality. Behav. Sci..

[5] Santabárbara J., Lasheras I., Lipnicki D.M., Bueno-Notivol J., Pérez-Moreno M., López-Antón R., De la Cámara C., Lobo A., Gracia-García P. (2021). Prevalence of anxiety in the COVID-19 pandemic: An updated meta-analysis of community-based studies. Prog. Neuropsychopharmacol. Biol. Psychiatry.

[6] Benfante A., Di Tella M., Romeo A., Castelli L. (2020). Traumatic Stress in Healthcare Workers During COVID-19 Pandemic: A Review of the Immediate Impact. Front. Psychol..

[7] Castiglioni M., Caldiroli C.L., Negri A., Manzoni G.M., Procaccia R. (2023). Linguistic Predictors of Psychological Adjustment in Healthcare Workers during the COVID-19 Pandemic. Int. J. Environ. Res. Public Health.

[8] Di Tella M., Benfante A., Castelli L., Romeo A. (2021). Anxiety, depression, and posttraumatic stress in nurses during the COVID-19 outbreak. Intensive Crit. Care Nurs..

[9] Vagni M., Maiorano T., Giostra V., Pajardi D. (2020). Coping With COVID-19: Emergency Stress, Secondary Trauma and Self-Efficacy in Healthcare and Emergency Workers in Italy. Front. Psychol..

[10] Zara G., Settanni M., Zuffranieri M., Veggi S., Castelli L. (2021). The long psychological shadow of COVID-19 upon healthcare workers: A global concern for action. J. Affect. Disord..

[11] Gutiérrez G., Barbarin O., Klicperová-Baker M., Padakannaya P., Thompson A., Crowe S., Khoury B. (2021). A Global Perspective on Psychologists’ and Their Organizations’ Response to a World Crisis. Interam. J. Psychol..

[12] Castiglioni M., Caldiroli C.L., Procaccia R., Conte F., Neimeyer R.A., Zamin C., Paladino A., Negri A. (2023). The Up-Side of the COVID-19 Pandemic: Are Core Belief Violation and Meaning Making Associated with Post-Traumatic Growth?. Int. J. Environ. Res. Public Health.

[13] Karekla M., Höfer S., Plantade-Gipch A., Neto D.D., Schjødt B., David D., Schütz C., Eleftheriou A., Pappová P.K., Lowet K. (2021). The role of psychologists in healthcare during the COVID-19 pandemic: Lessons learned and recommendations for the future. Eur. J. Psychol. Open.

[14] Aafjes-van Doorn K., Békés V., Luo X., Prout T.A., Hoffman L. (2022). Therapists’ resilience and posttraumatic growth during the COVID-19 pandemic. Psychol. Trauma.

[15] Houtsma C., Boffa J.W., Raines A.M., Constans J.I., Martin-Klinger C., Konur B.B., Franklin C.L., Jones K.L. (2022). Coping in crisis: The role of psychologists in response to a pandemic. Psychol. Serv..

[16] Chenneville T., Schwartz-Mette R. (2020). Ethical considerations for psychologists in the time of COVID-19. Am. Psychol..

[17] Serrão C., Rodrigues A.R., Teixeira A., Castro L., Duarte I. (2022). The impact of teleworking in psychologists during COVID-19: Burnout, depression, anxiety, and stress. Front. Public Health.

[18] Lin L., Stamm K.E., Ferenz K., Wright C.V., Bethune S., Conroy J. (2022). Relationship between challenges with the use of telehealth and psychologists’ response during the coronavirus pandemic. Prof. Psychol. Res. Pract..

[19] McLennan J., Marques M.D., Every D. (2020). Conceptualising and measuring psychological preparedness for disaster: The Psychological Preparedness for Disaster Threat Scale. Nat. Hazards.

[20] Reser J.P., Morrissey S.A. (2009). The crucial role of psychological preparedness for disasters. InPsych.

[21] Malkina-Pykh I.G., Pykh Y.A. (2013). An integrated model of psychological preparedness for threat and impacts of climate change disasters. WIT Trans. Built Environ..

[22] Freeman D., Bird J.C., Loe B.S., Kingdon D., Startup H., Clark D.M., Ehlers A., Černis E., Wingham G., Evans N. (2020). The Dunn Worry Questionnaire and the Paranoia Worries Questionnaire: New assessments of worry. Psychol. Med..

[23] Spitzer R.L., Kroenke K., Williams J.B., Löwe B. (2006). A brief measure for assessing generalized anxiety disorder: The GAD-7. Arch. Intern. Med..

[24] Humer E., Pammer B., Schaffler Y., Kothgassner O.D., Felnhofer A., Jesser A., Pieh C., Probst T. (2023). Comparison of mental health indicators in clinical psychologists with the general population during the COVID-19 pandemic. Sci. Rep..

[25] Vagni M., Maiorano T., Giostra V., Pajardi D., Bartone P. (2022). Emergency Stress, Hardiness, Coping Strategies and Burnout in Health Care and Emergency Response Workers During the COVID-19 Pandemic. Front. Psychol..

[26] Cerasa A., Craig F., Foti F., Palermo L., Costabile A. (2022). The impact of COVID-19 on psychologists’ practice: An Italian experience. J. Affect. Disord. Rep..

[27] Martiniuk A., Toepfer A., Lane-Brown A. (2023). A review of risks, adverse effects and mitigation strategies when delivering mental health services using telehealth. J. Ment. Health.

[28] Banerjee D.D. (2020). Psychological preparedness for the COVID-19 pandemic, perspectives from India. Psychiatry Res..

[29] Gandhi S., Sahu M., Govindan R., Gandhi S., Sudhir P.M., Balachandran R. (2021). Psychological preparedness for pandemic (COVID-19) management: Perceptions of nurses and nursing students in India. PLoS ONE.

[30] Zingela Z., van Wyk S., Bronkhorst A., Groves C. (2022). Developing a healthcare worker psychological preparedness support programme for the COVID-19 outbreak. S. Afr. J. Psychiatry.

[31] Said N.B., Molassiotis A., Chiang V.C.L. (2020). Psychological preparedness for disasters among nurses with disaster field experience: An international online survey. Int. J. Disaster Risk Reduct..

